# A potent and broad‐spectrum neutralizing nanobody for SARS‐CoV‐2 viruses, including all major Omicron strains

**DOI:** 10.1002/mco2.397

**Published:** 2023-10-26

**Authors:** Hebang Yao, Hongyang Wang, Zhaoyong Zhang, Yuchi Lu, Zhiying Zhang, Yu Zhang, Xinyi Xiong, Yanqun Wang, Zhizhi Wang, Haitao Yang, Jincun Zhao, Wenqing Xu

**Affiliations:** ^1^ School of Life Science and Technology ShanghaiTech University Shanghai China; ^2^ State Key Laboratory of Respiratory Disease National Clinical Research Center for Respiratory Disease Guangzhou Institute of Respiratory Health The First Affiliated Hospital of Guangzhou Medical University Guangzhou Guangdong China; ^3^ Shanghai Institute for Advanced Immunochemical Studies ShanghaiTech University Shanghai China; ^4^ GMU‐GIBH Joint School of Life Sciences Guangzhou Medical University Guangzhou Guangdong China; ^5^ Guangzhou Laboratory Bio‐Island Guangzhou Guangdong China; ^6^ Institute of Infectious Disease Guangzhou Eighth People's Hospital of Guangzhou Medical University Guangzhou Guangdong China; ^7^ Institute for Hepatology National Clinical Research Center for Infectious Disease, Shenzhen Third People's Hospital Shenzhen Guangdong China

**Keywords:** broad spectrum, nanobody, neutralize, Omicrons, SARS‐CoV‐2

## Abstract

SARS‐CoV‐2 viruses are highly transmissible and immune evasive. It is critical to develop broad‐spectrum prophylactic and therapeutic antibodies for potential future pandemics. Here, we used the phage display method to discover nanobodies (Nbs) for neutralizing SARS‐CoV‐2 viruses especially Omicron strains. The leading nanobody (Nb), namely, Nb4, with excellent physicochemical properties, can neutralize Delta and Omicron subtypes, including BA.1, BA.1.1 (BA.1 + R346K), BA.2, BA.5, BQ.1, and XBB.1. The crystal structure of Nb4 in complex with the receptor‐binding domain (RBD) of BA.1 Spike protein reveals that Nb4 interacts with an epitope on the RBD overlapping with the receptor‐binding motif, and thus competes with angiotensin‐converting enzyme 2 (ACE2) binding. Nb4 is expected to be effective for neutralizing most recent Omicron variants, since the epitopes are evolutionarily conserved among them. Indeed, trivalent Nb4 interacts with the XBB1.5 Spike protein with low nM affinity and competes for ACE2 binding. Prophylactic and therapeutic experiments in mice indicated that Nb4 could reduce the Omicron virus loads in the lung. In particular, in prophylactic experiments, intranasal administration of multivalent Nb4 completely protected mice from Omicron infection. Taken together, these results demonstrated that Nb4 could serve as a potent and broad‐spectrum prophylactic and therapeutic Nb for COVID‐19.

## Graphical abstract

The leading nanobody (Nb), namely, Nb4, with excellent physicochemical properties, can neutralize Delta and Omicron subtypes including the major Omicrons, and is expected to be effective for neutralizing most recent Omicron variants, including BF.7, BQ1.1, and XBB1.5. The crystal and Cryo‐EM structure uncover an epitope, which is evolutionarily conserved among Omicrons. Prophylactic and therapeutic experiments in mice indicated that Nb4 could reduce the Omicron virus loads in the lung. In particular, in prophylactic experiments, intranasal administration of multivalent Nb4 (Nb4‐16t) completely protected mice from Omicron infection.

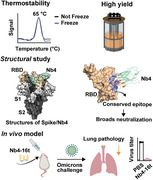

## 1 INTRODUCTION

Different variants of concern (VOCs) of severe acute respiratory syndrome coronavirus 2 (SARS‐CoV‐2), especially the Omicron variants, remain a global health threat. The SARS‐CoV‐2 spike (S) protein is not only responsible for host cell entry but also the primary antigen for antibody generation in the human body elicited by vaccine or infection.^1^, ^2^ Mutations in the S protein associated with recent Omicron variants maintained or even enhanced tight binding of the spike protein to angiotensin‐converting enzyme 2 (ACE2),^3^, ^4^ which resulted in immune escape and rounds of the infection.^2^ Different Omicron sublineages, including BQ.1/BQ1.1, XBB.1.5, and XBB.1.16, have emerged and may continue to appear one after another, spreading worldwide at an even faster pace compared with previous variants.^5^

The remarkable resistance of Omicron variants to neutralizing antibodies and sera from previously vaccinated or infected persons has become a major clinical concern.^2^, ^6^, ^7^, ^8^ Although with reduced protection efficiency of most antibodies and vaccines for new Omicron variants,^9^, ^10^, ^11^, ^12^, ^13^ neutralizing antibody treatment still protects many infected people from severe symptoms and death. Although efficacious drugs have been developed to treat COVID‐19,^14^, ^15^, ^16^, ^17^, ^18^, ^19^ developing new broad‐spectrum antibodies against emerging coronavirus strains remains urgent and necessary.^20^

Compared to conventional antibodies, nanobodies (Nbs) have many advantages, including high thermostability, great production efficiency, low cost, and easy engineering.^21^, ^22^, ^23^, ^24^ Nbs could be separated from immunized camelids or screened from a synthetic library.^25^ Although a number of antibodies against SARS‐CoV‐2 receptor‐binding domains (RBDs) have been discovered and approved,^9^, ^10^, ^11^, ^12^, ^13^ most of them face the problem of immune escape of new Omicron strains.^26^, ^27^ Therefore, discovery of new broad‐spectrum Omicron neutralizing Nbs is still highly valuable for fighting emerging and future COVID‐19 pandemics.

Here, we report a potent and thermostable Nb, namely, Nb4, which can neutralize all of the Omicron subvariants at comparable levels. Structural analysis of Omicron S protein or RBD in complex with Nb4 suggests that Nb4 has a unique and critical epitope for the Omicrons that is evolutionarily conserved. Along with cell‐ and mouse‐based viral infection assay results, we conclude that Nb4 is a useful broad‐spectrum neutralizing Nb against emerging Omicron variants.

## 2 RESULTS

### 2.1 Discovery and characterization of Nbs targeting SARS‐CoV‐2 Omicron

**FIGURE 1:**
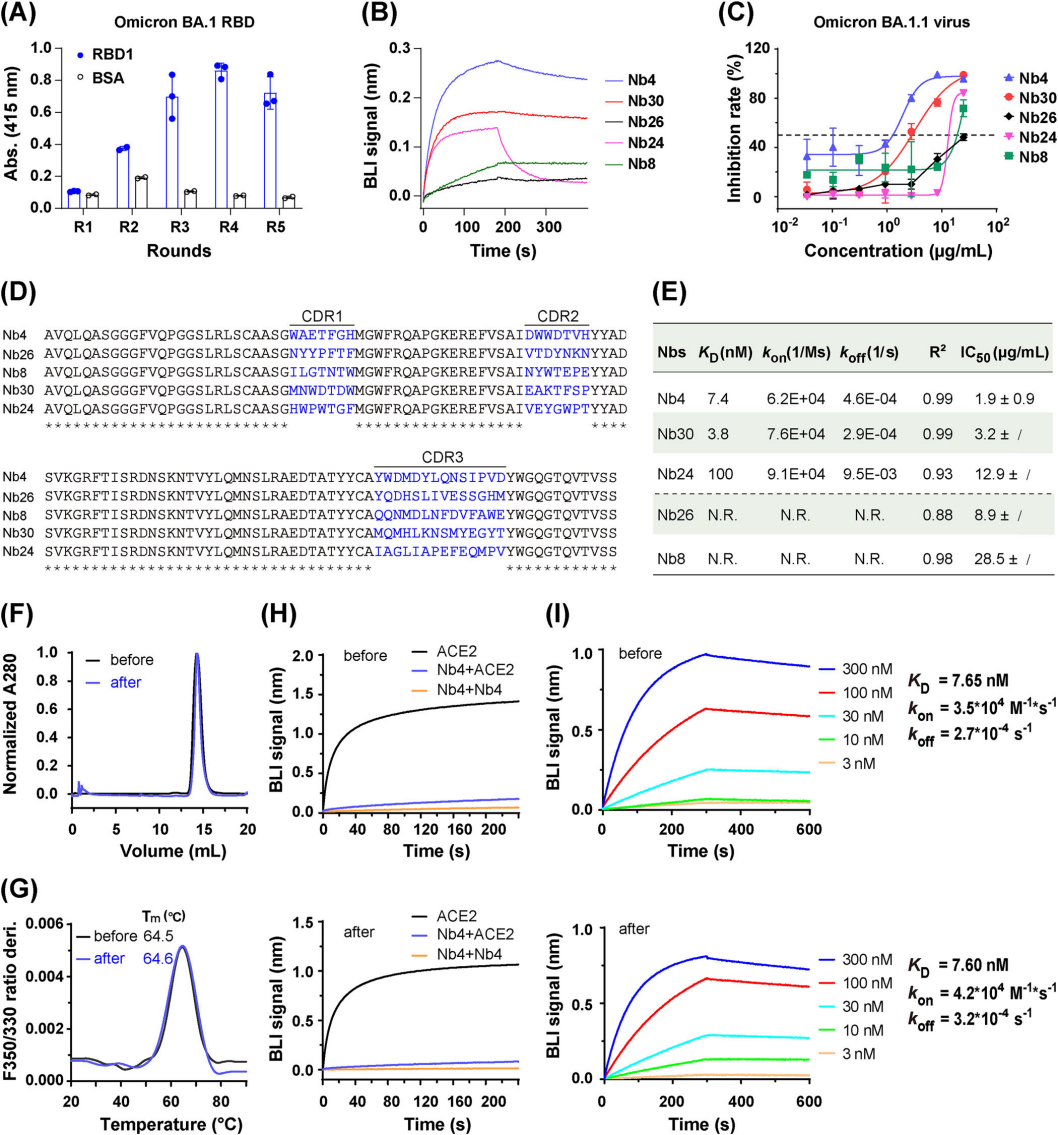
The identification and characterization of nanobodies (Nbs) neutralizing against BA.1.1. (A) Polyclonal enzyme‐linked immunosorbent assay (ELISA) shows the enrichment of the phages binding to receptor‐binding domain 1 (RBD1) after each round of panning. Blue column represents RBD1 coated wells, and the black represents BSA background binding. The round number and RBD1 concentration were R1 (700 nM), R2 (700 nM), R3 (100 nM), R4 (100 nM), and R5 (10 nM). (B) Biolayer interferometry (BLI) assay to confirm the binding of purified Nbs to RBD1 in vitro, the represented curves are shown. (C) The neutralization assay was performed using BA.1.1 virus. (D) Sequence alignment of the neutralization Nbs. CDR1/2/3, complementarity determining region 1/2/3. (E) Summary of the binding kinetics parameters between Nbs and RBD1 and neutralization IC_50_. (F) SEC analysis of Nb4 before (black) and after (blue) lyophilization. (G) Differential scanning fluorometry (DSF) profiles of the Nb4 before (black) and after (blue) lyophilization show changes in its intrinsic fluorescence (the first derivative of the fluorescence ratio at 350/330 nm) as a function of temperature. (H) Competition assay to evaluate the ability to compete with the angiotensin‐converting enzyme 2 (ACE2) binding (upper and lower panels). (I) Binding kinetics of RBD1 and Nb4 was determined by BLI (upper and lower panels). N.R., non‐reliable results due to the unreliable dissociation data.

To identify neutralizing Nbs against SARS‐CoV‐2 Omicron strains, BA.1 RBD (hereafter referred to as RBD1) was used as the antigen for selection against a phage library. After five rounds of phage panning, polyclonal and monoclonal enzyme‐linked immunosorbent assays (ELISAs) were carried out to evaluate the enrichment (Figure 1A) and identify the Nbs binding to RBD1, respectively. After sequencing, 35 unique Nbs were obtained, further purified, and used in a biolayer interferometry (BLI) assay to determine the binding affinities of Nbs to RBD1. BLI assay suggested that 19 out of 35 Nbs bind to RBD1 (Figures 1B and S1 and Table S1). Then, an authentic virus challenge cell assay was used to evaluate neutralization activities, which demonstrated that five out of these 19 Nbs have decent neutralization activity (Figure 1C). Sequence alignment, binding kinetics and half‐maximum inhibitory concentration (IC_50_) of these five Nbs are summarized in Figure 1D,E. Among them, Nb4 and Nb30 have greater potential for prophylactic and therapeutic applications than other Nbs. The leading Nb (Nb4) has an IC_50_ value of about 1.9 ± 0.9 μg/mL.

Nb4 has excellent solubility (>10 mg/mL in phosphate‐buffered saline [PBS]) and thermal stability. To evaluate physicochemical properties, the tolerance of Nb4 to lyophilization was performed in four aspects, including homogeneity (Figure 1F), thermostability (Figure 1G), binding competition ability against ACE2 (Figure 1H), and binding affinities with RBD1 (Figure 1I). These results suggest that lyophilization has no detectable effect on the physicochemical characteristics of Nb4, showing that Nb4 would be suitable for lyophilization for easy storage and shipping.

### 2.2 Multivalency engineering of Nbs to enhance the binding affinity and neutralizing activity

**FIGURE 2:**
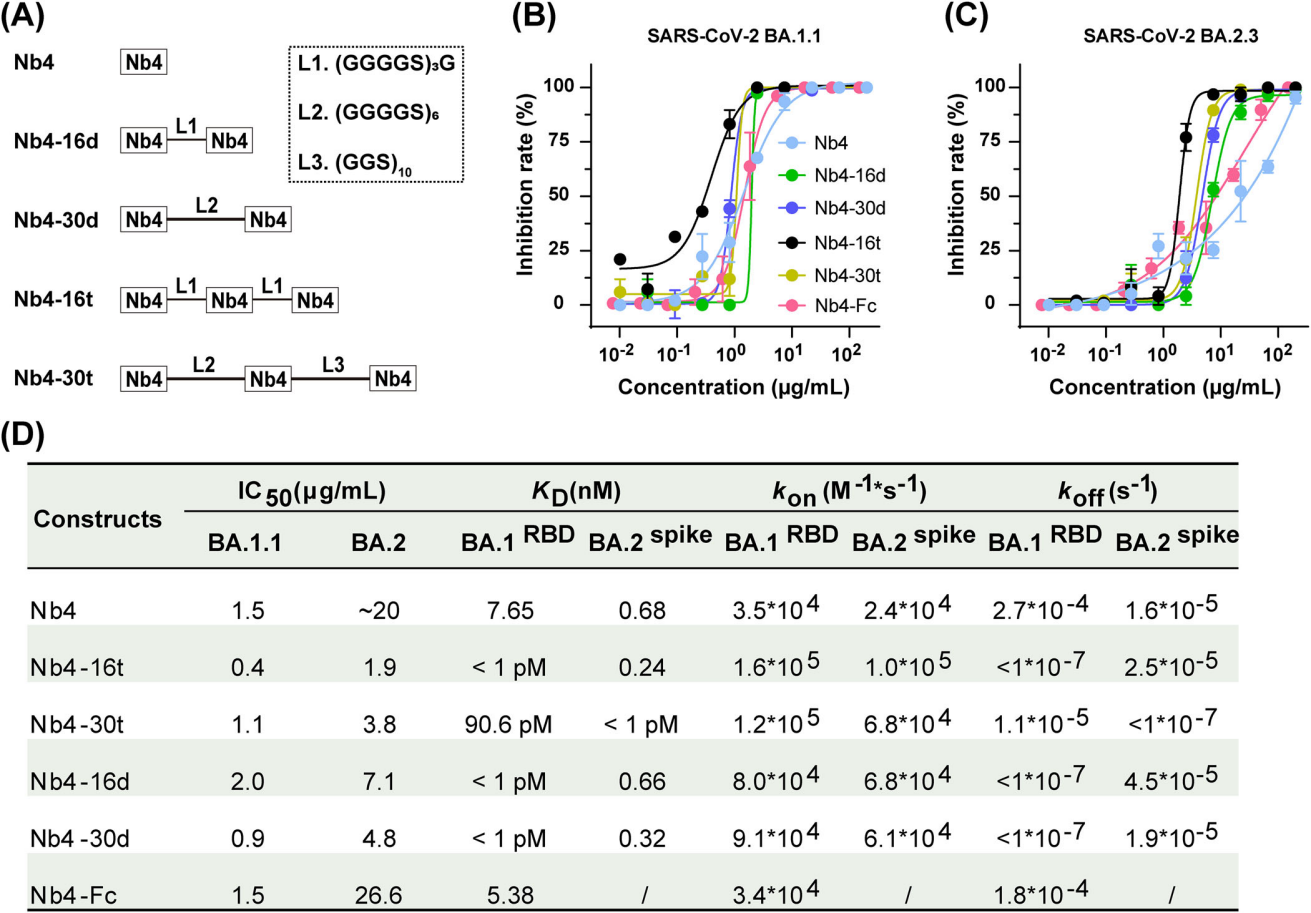
Evaluation of biochemical and neutralization properties of multivalent Nb4. (A) Schematics of the multivalent Nb4. (B and C) The challenge assay of different formats of Nb4 to BA.1.1 (B) and BA.2.3 (C) authentic virus in Vero E6 cells. (D) Summary of the binding kinetics and IC_50_ of the multivalent nanobodies (Nbs) against SARS‐CoV‐2.

To further improve the binding avidity and activity, we examined tandem multivalent Nb4 or Nb30 (bivalent and trivalent) protein constructs with flexible polypeptide linkers composed of glycine and serine (Figures 2A and S2). While monomeric Nb4 has a *K*_d_ of ∼8 nM with the RBD1, both the bivalent and trivalent Nb4s have dissociation constants with RBD1 in the pM range (Figure S3A). Additionally, different forms of Nb4, including the Nb4 monomer, could also bind to BA.2 spike extracellular domain (ECD), which contains three RBDs, with sub‐nM affinities (Figure S3B). In comparison, with the same assay condition, the affinity between RBD1 and hACE2 was determined to be ∼9 nM (Figure S3A). These results suggested that the different forms of Nb4 might neutralize the BA.1 and BA.2 viruses via direct competition with ACE2 binding (see below), and multivalent Nb4s are particularly efficient in S protein binding.

To evaluate the viral inhibition activities, we used the authentic BA.1.1 and BA.2.3 Omicron viruses in a challenge assay in Vero E6 cells (Figure 2B,C). For BA.1.1, different forms of Nb4 have different neutralization activities, ranging from 0.4 to 2.0 μg/mL, and the Nb4‐16t form (with three tandem Nb4s connected by two 16‐residue linkers) is the most potent one, reaching up to 0.4 μg/mL. For BA.2.3, the largest difference in the IC_50_ between different Nb4 constructs was about 10‐fold, and the best one was also Nb4‐16t, with neutralization activity of 1.9 μg/mL (Figure 2D). Meanwhile, the neutralization activity of the Nb4‐Fc fusion protein was also evaluated, which was similar to that of monomer Nb4 (Figure 2B–D). In addition to Omicron strains, Nb4‐16t could also neutralize the Delta strain (B.1.617.2) with an IC_50_ value of ∼22 μg/mL (Figure S4).

### 2.3 Prophylactic and therapeutic efficacy of Nb4 against SARS‐CoV‐2 infection in mice

**FIGURE 3:**
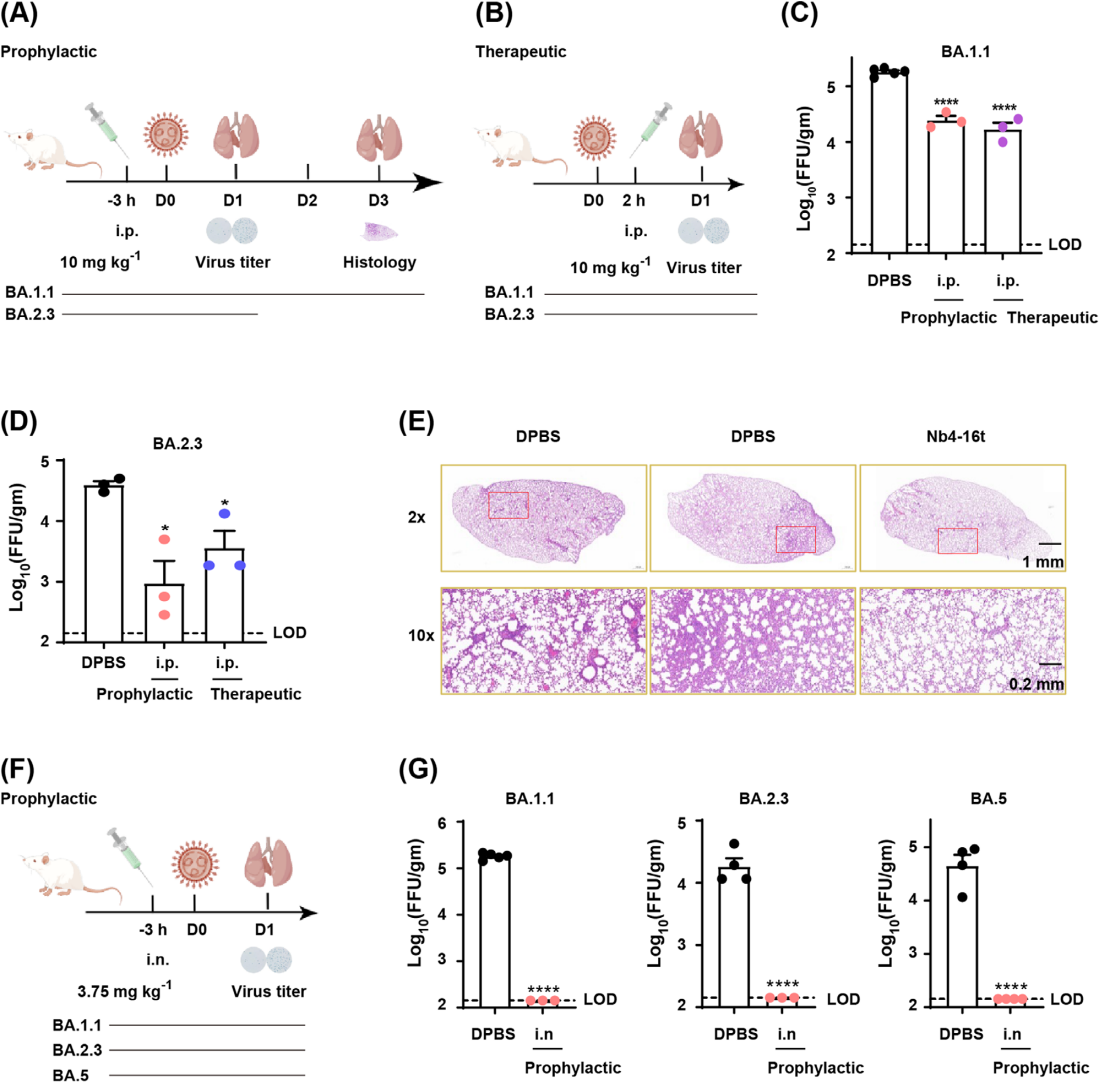
Nb4‐16t provides prophylactic and therapeutic protection in BA.1.1‐, BA.2.3‐, and BA.5‐infected mice. (A and B) Animal experimental design schemes for prophylactic (A) and therapeutic (B) effects with intraperitoneal (i.p.). (C and D) Quantification of lung virus titers after the mice were sacrificed. (E) Histopathological analysis of lungs from infected mice. Lungs were harvested and stained with hematoxylin and eosin (H&E). Scale bars, upper panel (1 mm) and lower panel (200 μm), which scale up the red box in the upper panel. Representative images were from each group. (F) Experimental design scheme for prophylactic effects from BA.1.1, BA.2.3, and BA.5 virus challenge in mice with intranasal (i.n.) administration. (G) Quantification of the titers of virus in lung. Data of BA.1.1 in Figure 3C,G were from the same experiments, so have same DPBS control as indicated. *n* ≥ 3. Statistical significance was tested by unpaired *t*‐test. LOD, limit of detection. ^*^ *p* ≤ 0.05, ^**^ *p* ≤ 0.01, ^***^ *p* ≤ 0.001, ^****^ *p* ≤ 0.0001.

To evaluate the protection effect of Nb4‐16t against SARS‐CoV‐2 Omicron BA.1.1, BA.2.3, and BA.5 challenge with intraperitoneal (i.p.) (Figure 3A,B) or intranasal (i.n.) (Figure 3F) administration, an authentic virus challenge experiment was performed in a biosafety level 3 (BSL‐3) setting. Briefly, wild‐type BALB/c mice were used for natural infection by Omicron strains in this experiment, as previously reported for Omicron BA.1.1 and BA.2.3 infection experiments in BALB/c mice.^5^, ^28^ After viral infection, the lungs were harvested at indicated time points, and the virus titer was quantified using a focus‐forming assay (FFA) and reported as focus‐forming units (FFUs) per gram lung. As shown in Figure 3C,D, both prophylactic and therapeutic treatment intraperitoneally (10 mg/kg) demonstrated a significant reduction in lung virus in comparison to the Dulbecco's phosphate‐buffered saline (DPBS) control group. As shown by histopathological analysis of the lungs, pre‐administration intraperitoneally of Nb4‐16t could inhibit perivascular and parenchymal infiltration evidently compared with the control group (Figure 3E).

Strikingly, intranasal administration of Nb4‐16t (3.75 mg/kg) in mice provided much more potent protection against Omicron infections than intraperitoneal administration (Figure 3G). Indeed, after intranasal administration of Nb4‐16t at a low dose of 3.75 mg/kg, virus levels in mice infected with Omicron BA.1.1, BA.2.3, and BA.5 strains, respectively, all stayed within the lowest viral detection limit in the FFAs (Figure 3G). Taken together, our data indicate that Nb4‐16t can provide strong protection against the three Omicron subvariants, especially intranasal administration, reaching to the virus detection limit.

### 2.4 Crystal structure of the Nb4–RBD1 complex and neutralization mechanism

**FIGURE 4:**
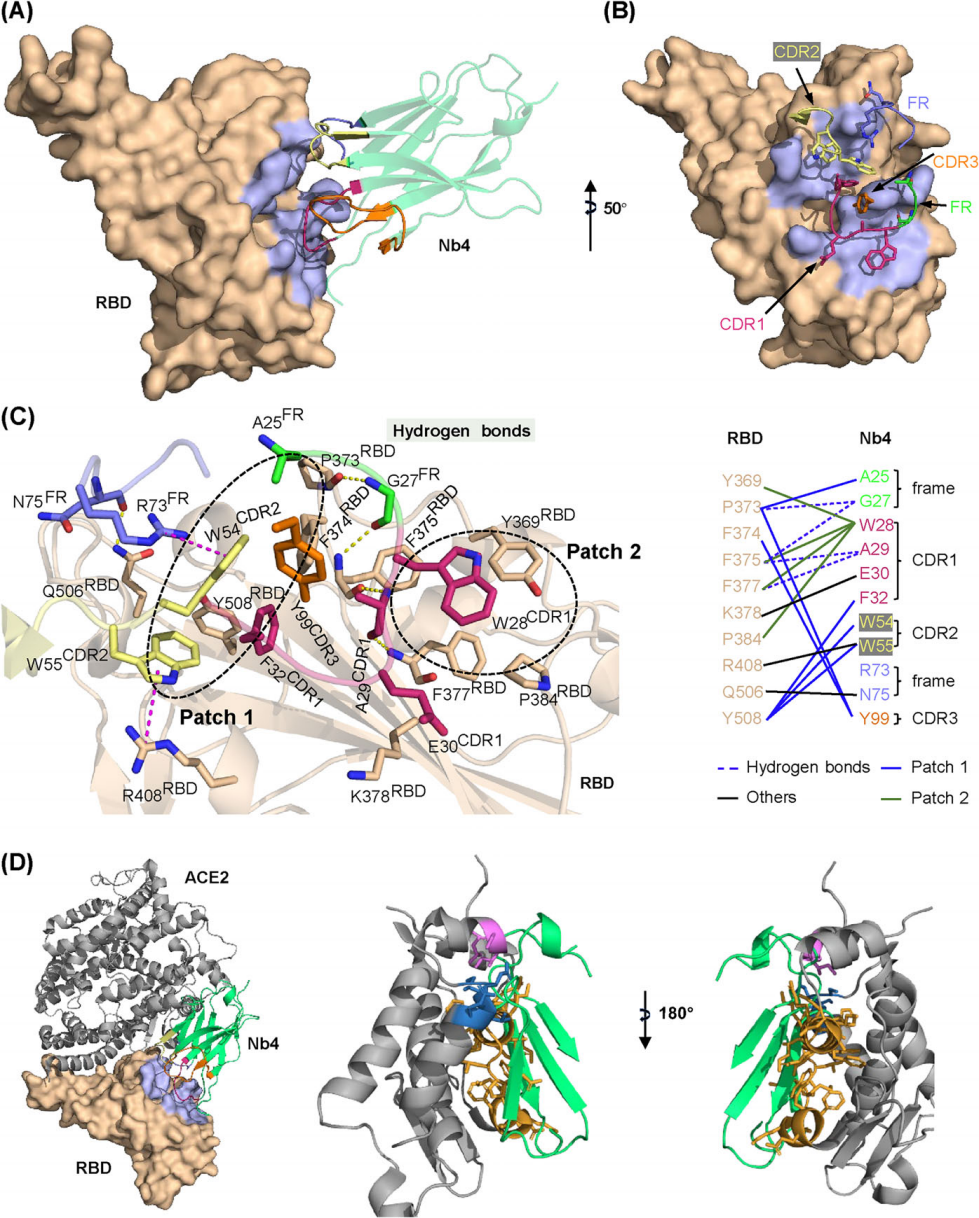
Structural basis of Nb4 interaction with receptor‐binding domain 1 (RBD1) and neutralization. (A) Interface between Nb4 and RBD1. The epitope (light blue) on the RBD (wheat) and Nb4 (green) with colored interface regions including complementarity determining region (CDR)1 (red), CDR2 (yellow), CDR3 (orange), and framework (FR) (blue and green) are shown. (B) Stick model for the interface region of Nb4 and RBD1, and the color code as (A). (C) Hydrophobic network (Patch 1), which is on the left of the hydrogen bonds, is composed of CDR1/2/3 and FR. The hydrogen bonds and another hydrophobic network (Patch 2) are shown on the right of Patch 1. The right panel shows the residues involved in the interaction. (D) Superposition of RBD1‐Nb4 complex to angiotensin‐converting enzyme 2 (ACE2)‐RBD1 complex (PDB: 7WBP). Nb4 clashes with ACE2 on three regions, consisting of 303−329 (orange), 420−423 (magenta), and 545−549 (blue). These are shown in two angles as indicated.

To understand the molecular mechanism of Nb4 neutralizing effect, crystal structure of the BA.1 RBD (RBD1 for short) in complex with Nb4 was determined at 2.4 Å resolution (Figure 4A and Table S2). According to the previously defined classification of neutralization Nbs,^29^ Nb4 could be classified in class II, which binds to a conserved RBD surface area of β‐coronaviruses.^29^ RBD1 interacts with both Nb4 complementarity determining regions (CDRs) and the Nb4 framework (FR), with Nb4 CDR1, instead of its CDR3, making major contacts.^29^ This RBD1–Nb4 interface is formed by two hydrophobic patches and a hydrogen bond network (Figure 4B,C). The first hydrophobic patch is composed of F32^CDR1^, W54^CDR2^, W55^CDR2^, Y508^RBD^, F374^RDB^, Y99^CDR3^, and A25^FR^, with F32^CDR1^ inserting into a RBD1 groove (Figure 4B,C). This patch is enhanced by a hydrogen bond between N75^FR^ and Q506^RDB^ and two cation‐π interactions of W54^CDR2^ and R73^FR^ and W55^CDR2^ and R408^RBD^ (Figure 4C). In the second hydrophobic patch, Nb4 residue W28^CDR1^ is engaged in a hydrophobic network, including four RBD residues (Y369, F375, F377, and P384) (Figure 4C). Importantly, mainchain amine and carbonyl groups of Nb4 G27^FR^ and A29^CDR1^ form a network of hydrogen bonds with the mainchain atoms of P373^RBD^, F375^RBD^, and F377^RBD^, by which the two hydrophobic patches are connected and enhanced (Figure 4C). Thus, our crystal structure of RBD1–Nb4 complex provides a solid foundation for understanding the specific and strong interaction between RBD1 and Nb4.

In our structure (Figure 4A), the epitope of Nb4 on RBD1 partially overlaps with RBD1's interface with ACE2, sharing three residues in the receptor‐binding motif (residues numbers of 438−506) of RBD1.^30^, ^31^ Superposition of our RBD1–Nb4 complex crystal structure with previously reported RBD1–ACE2 complex structure^32^ (Protein Data Bank [PDB]: 7WBP) demonstrates that, for RBD1 binding, ACE2 strongly clashes with the FR or CDR2 of Nb4, indicating neutralization via direct competition for RBD1 binding (Figure 4D).

**FIGURE 5:**
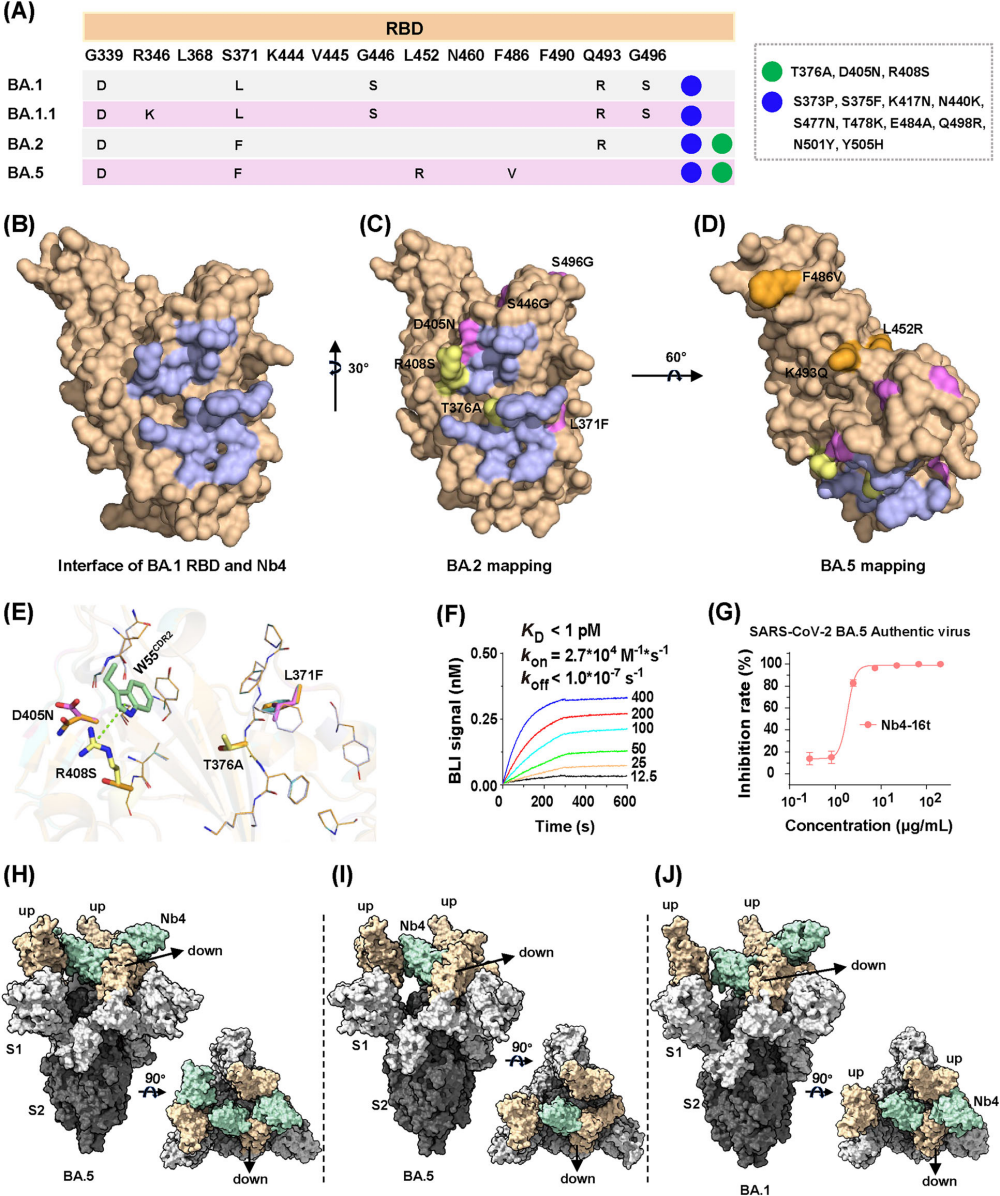
Structural analysis of the Nb4 neutralizing against different Omicron variants. (A) Mutations in the receptor‐binding domains (RBDs) of different Omicron strains. (B) Epitope mapping of Nb4 on RBD1 (light blue). (C) Mapping the differences in six residues (magenta and yellow) between BA.1 and BA.2 based on epitope of Nb4. (D) Mapping the differences of three residues (orange) between BA.2 and BA.5. (E) Superposition of the RBD1 and Swiss model of RBD2 (RBD domain of BA.2) in complex with Nb4. The four residues (376, 371, 405, and 408), which may affect the binding between RBD2 and Nb4, are shown as sticks. (F) Biolayer interferometry (BLI) assay to determine the binding between BA.5 Spike and Nb4, the concentration (nM) is shown as indicated. (G) Authentic virus neutralization assay of Nb4‐16t to BA.5. (H and I) The Cryo‐EM surface structure of the Nb4‐16t in complex with BA.5 Spike was observed in two classes (H, 3.5 Å, three Nb4s bound with BA.5 Spike in the “2‐RBD up/1‐RBD down” conformation; I, 3.3 Å, one Nb4 bound with BA.5 Spike “down” RBD in the “2‐RBD up/1‐RBD down” conformation). (J) The Cryo‐EM surface structure of the Nb4‐30t in complex with BA.1 Spike was observed in one class (3.6 Å, two Nb4s bound with BA.1 Spike in the “2‐RBD up/1‐RBD down” conformation).

Nb4 was initially screened using the BA.1 RBD. The RBDs of BA.1 and BA.2 are different in six residues (Figure 5A–C), which appeared to have a minor impact on Nb4 binding and neutralization activities (Figure 2D). Among these six BA.2 residues, mutations of S446G and S496G occur far away from the Nb4‐binding epitope and thus are not expected to have any impact on the interaction. Although L371F and D405N are close to the epitope, their sidechains are pointing away from the interface (Figure 5E). For the last two residues, the T376 sidechain is not directly involved in binding, and structural modeling suggests that the T376A mutation may have little effect on the formation of the hydrogen bonds among F375/F377 mainchain atoms with Nb4. The last R408S mutation would likely disrupt the cation‐π interaction as indicated (green dash, Figure 5E). This could be one of the possible reasons for moderately weakened neutralization capability of Nb4 monomer to BA.2 compared to BA.1.1 (Figure 2D). Thus, our crystal structure explains how multivalent Nb4, such as Nb4‐16t and Nb4‐30t, can efficiently neutralize both BA.1 and BA.2 (Figure 2D), among other newly emerging Omicron variants (see below).

### 2.5 Cryo‐EM structures of trivalent Nb4 in complex with BA.1, BA.5 S protein reveals its inhibitory mode

Compared to BA.2, BA.5 has three different residues in the RBD region (Figures 5A and S5), which are all away from the Nb4 binding epitope and thus not expected to interfere with Nb4 binding (Figure 5A,D). In order to confirm the interaction between Nb4 and BA.5, a BLI assay was carried out and we found that the binding between Nb4‐16t and BA.5 S protein has a sub‐pM *K*_d_ with no detectable dissociation (Figure 5F). Consistently, the authentic virus assay showed that Nb4‐16t can neutralize BA.5 with a neutralization capacity of ∼1.8 μg/mL (Figure 5G).

To understand the neutralization mechanism of Nb4 to Omicron viruses, we used single‐particle Cryo‐EM method to solve the 3D structure of Nb4‐16t in complex with the BA.5 S protein, as well as that of the BA.1 S protein in complex with Nb4‐30t (Table S3). For BA.5 S protein in complex with Nb4‐16t, a “2‐RBD up/1‐RBD down” conformation of the Spike protein was dominant (Figures 5H,I and S6A). Corresponding particles could be further classified into two classes. We solved the structure of the first class with three Nb4s binding three RBDs at 3.5 Å resolution (Figures 5H and S6C), and the second class with only one Nb4 on the “down” RBD at 3.3 Å resolution (Figures 5I and S6B). As for the BA.1 S protein in complex with Nb4‐30t, we also observed a “2‐RBD up/1‐RBD down” conformation of S protein with two copies of Nb4s at 3.6 Å resolution, with one copy of Nb4 on the “down” RBD and the other Nb4 on one of the “up” RBDs (Figures 5J and S6D,E). In all three Cryo‐EM structures, the Nb4‐S protein interfaces have significantly lower local resolutions (Figure S6), which do not clearly show densities for Nb sidechains but allow us to build Nbs with confidence due to our high‐resolution RBD–Nb4 crystal structure (see above).

Nb4 can bind RBDs in both up and down positions using the same RBD–Nb4 interface. The Nb4 unit binding to the RBD in down position appears to be more stable than those in up positions (Figure 5H–J), likely because of additional van der Waals contacts formed between the Nb4 in down position and one of the two other RBDs in up positions (Figure 5H–J; detailed contacts remain unclear due to low local resolution). Interestingly, the Nb4 in down position would collide with the RBD–RBD packing interactions in the “all‐down” conformation of trimeric S protein (RBD1 numbering 371−376).^4^ Thus, binding of a Nb4 with one RBD unit in down position would block the RBD–RBD packing in the “all‐down” conformation and thus keep two other RBDs in up positions and expose more epitopes. We suggest that via binding of Nb4 to one of the RBDs in S protein trimer, other RBD‐binding antibodies or Nbs, such as another Nb4 unit in a multivalent Nb4, can then bind to the exposed RBDs to enhance the neutralization capacity.

### 2.6 Structural basis of broad‐spectrum neutralization efficiency of Nb4 for emerging Omicron variants

**FIGURE 6:**
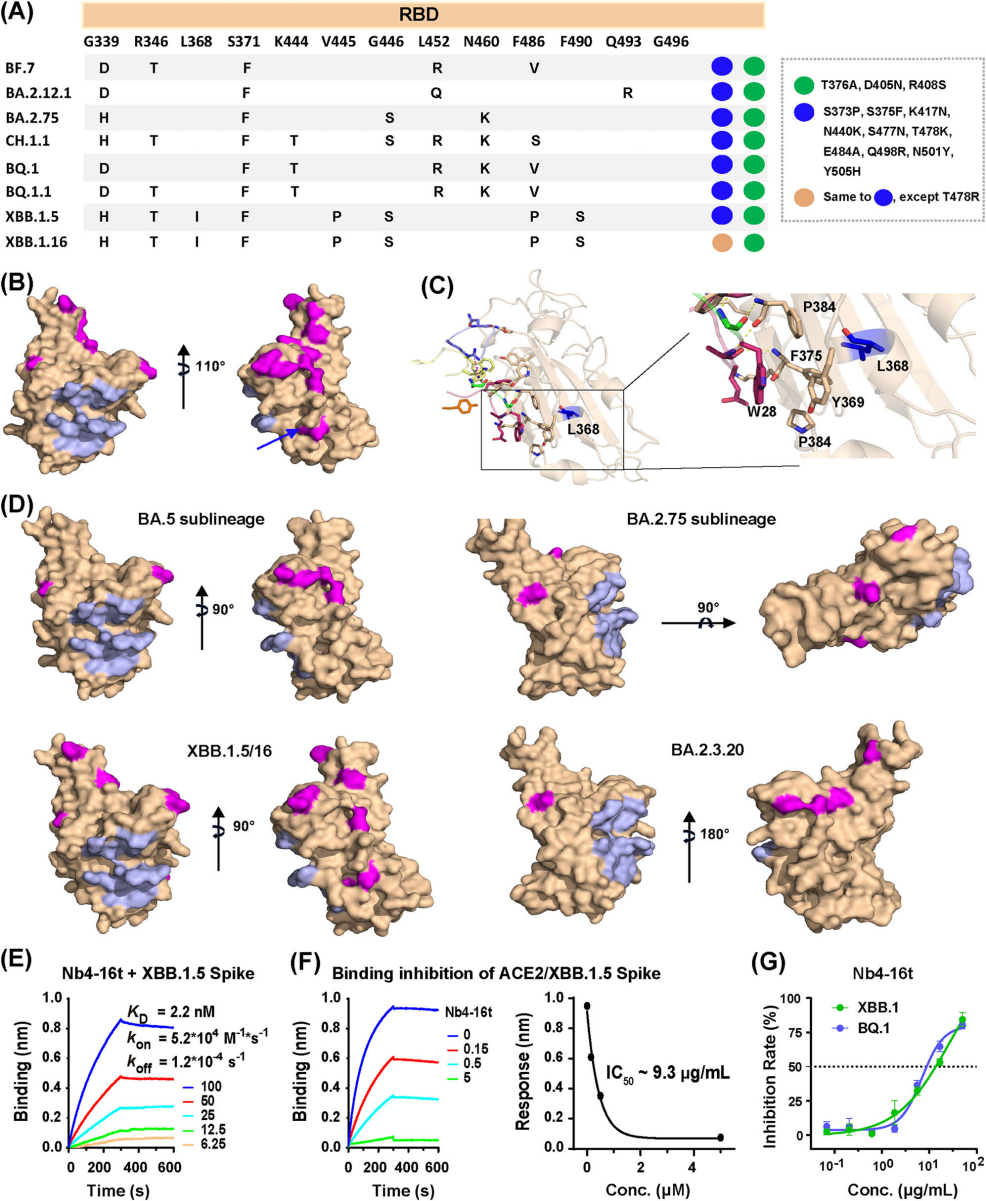
Mutation mapping of newly emerging Omicron subvariants in the receptor‐binding domain (RBD). (A) Mutations in the RBDs of different Omicron strains. (B) The mutations on RBDs of the Omicron subvariants under monitoring were mapped, and showed that the mutations (magenta) were away from the Nb4 epitope (light blue). (C) The L368 is shown to clarify the distance from the Nb4 epitope. (D) The mutations in each subvariant were mapped on the RBD, showing the relationship with Nb4 epitope. (E) Binding kinetics between Nb4‐16t and XBB.1.5 Spike protein. (F) A competition biolayer interferometry (BLI) assay was carried out to determine the binding inhibition of 50 nM XBB.1.5 Spike to ACE2, which was immobilized on the SA sensor, by Nb4‐16t at various concentrations (μM). The apparent IC_50_ (∼ 200 nM/9.3 μg/mL) of Nb4‐16t is shown as the fitted curve (right panel). (G) Authentic virus neutralization assay of Nb4‐16t against XBB.1 and BQ.1.

To understand if Nb4 could also neutralize emerging new Omicron subvariants, including XBB.1.5 and XBB.1.16 (https://www.who.int/activities/tracking‐SARS‐CoV‐2‐variants) (Figure 6A), we mapped the variant residues to our structures as we did with BA.2 and BA.5 (Figure 6B–D). We found that in all key emerging new Omicron subvariants, all mutated residues were away from the Nb4 epitope except L368I (Figure 6B,C). Although this residue was close to Y369 involved in the hydrophobic Patch 2, it is unlikely to interfere with the interaction between Nb4 and RBDs due to the similarity of these two hydrophobic sidechains.

Indeed, purified Nb4‐16t can bind to purified XBB.1.5 Spike protein with a *K*_d_ of 2.2 nM (Figure 6E) and compete for the binding of ACE2 extracellular domain with the XBB.1.5 S protein of 50 nM, with an IC_50_ of 200 nM (9.3 μg/mL) (Figure 6F). This indicates that Nb4‐16t can be useful for neutralizing XBB1.5. The newly emerging XBB.1.16 has a mutation of T478R in the RBD region, whereas most Omicron VOCs, including XBB.1.5, carry a T478K mutation (Figure 6A). Since T478 is away from Nb4 epitope and T478K mutation has no effect on BA.5 neutralization, we expect that the binding and neutralization capacities of Nb4 to XBB.1.16 should be similar to those of XBB.1.5.

To test above analysis, we also performed live virus challenge assays of Nb4‐16t with BQ.1 and XBB.1, and observed IC_50_ values of 9 and 14 μg/mL, respectively (Figure 6G). Taken together, Nb4‐16t could neutralize Delta, BA.1, BA.1.1 (BA.1 + R346K), BA.2, BA.5, BQ.1, and XBB.1 using live virus with IC_50_ values ranging from 0.4 to 22 μg/mL. Based on our structural analysis, in vitro biochemical assays, virus titration and challenge assays, combined with relatively conserved binding epitopes on the S protein surfaces (Figures 5, 6, and S5), we conclude that Nb4 and its multivalent forms are broad‐spectrum Nbs for neutralizing SARS‐CoV‐2 VOCs, in particular Omicron strains. In addition, the newly emerging variant of EG.5 has an additional mutation of F456L, which is away from the Nb4 epitope, compared to XBB.1.5. It is likely that EG.5 strain could also be neutralized by Nb4‐16t.

## 3 DISCUSSION

The Omicron family of SARS‐CoV‐2 has many different strains, including BA.1, BA.1.1, BA.2, BA.3, BA.5, BF, BQ, CH.1.1, XBB.1.5, and XBB.1.16. The accumulated mutations in the RBD regions have caused severe antibody evasion and immune escape.^26^, ^27^ New and broad‐spectrum neutralization Nbs are urgently needed to fight ongoing and future SARS‐CoV‐2 pandemics.

Here, we initially selected and identified five Nbs from a synthetic library that could neutralize the BA.1.1 strain. Among them, Nb4 has a broad neutralization activity and excellent physicochemical properties. The critical residues involved in the interaction between RBD1 and Nb4 are relatively conserved in Alpha, Beta, Gamma, Delta, and Omicrons. Despite of substitutions in five critical residues (371, 373, 375, 376, and 408, Figure S5) among Delta and Omicron strains, trivalent Nb4 can still effectively bind to Delta S protein (Figure S4). Importantly, these residues are also involved in forming the interfacing loop of RBD (RBD1 numbering 371−376), which are important residues to inter‐RBD packing of Omicron variants. The inter‐RBD packing, which involves extensive hydrophobic and hydrogen‐bond interactions, stabilizes the RBDs so that the S protein trimer is more likely to stay in the down conformations without impairing the affinity to ACE2 when RBD is in an up position.^4^ Residues involved in inter‐RBD packing are evolutionarily conserved. Our Nb4 achieves broad‐spectrum binding activity to RBDs in part by binding to this conserved epitope on RBD.

We noticed that the neutralization capacity of Nb4 was not as impressive as that of some reported Nbs such as the monomer of C5G2^33^ or 3‐2A2‐4,^34^ the biparatopic Nbs of PiN‐31^35^ or aRBD‐2‐5‐Fc,^31^ and IgM format of R14 (MR14),^36^ all of which have single‐ to three‐digit nanograms per milliliter. However, as the authors described, C5G2 has no effect on the Delta strain caused by L452R mutation, which exists in the BA.4/5, BF.7, BQ.1, and BQ.1.1 strains.^33^ Thus, C5G2 might not broadly neutralize omicron variants. As for other Nbs mentioned above, their epitopes all have mutations in new Omicron variants, so further experiments are needed to confirm whether they can broadly neutralize these Omicron variants. Our analysis suggests that the Nb4 has the potential to neutralize all strains listed in Figure S5. It is noteworthy that TR14 (trimer of R14) to MR14 (IgM format of R14) increases the IC_50_ by ∼440‐fold,^36^ so it would be interesting to further test the IgM format of Nb4 in the future to improve the neutralization capacity.

As a therapeutic drug to replace conventional antibodies, Nbs still have some limitations, such as short half‐life and no non‐neutralizing activities. To elongate the half‐life of Nbs, many ways could be tested, such as fusing Fc to Nbs to increase the half‐life and improve the antiviral effect.^37^ At the same time, by engaging complement and Fc receptor‐containing immune cells, Fc fusion can also contribute to non‐neutralizing activities, such as complement‐dependent cytotoxicity and antibody‐dependent cellular cytotoxicity.^38^ Many studies suggested that the non‐neutralizing activities also play an important role against the SARS‐CoV‐2 infection.^38^, ^39^, ^40^ As the Nb4‐Fc (wild‐type IgG1) fusion tested in this study showed relatively weak in vitro neutralization effect (Figure 2B–D), the in vivo effect of Nb4‐Fc was not further evaluated. By using engineered Fc instead of wild‐type IgG1 Fc, the non‐neutralization activities protected mice from SARS‐CoV‐2.^39^ Thus, Fc engineering could be performed to apply the non‐neutralization activity to Nb4 in later investigations.

Collectively, we discovered a broad‐spectrum neutralizing Nb (Nb4), which may work synergistically with different Nbs that target other exposed epitopes to neutralize the SARS‐CoV‐2 virus. Although we now have small‐molecule drugs for COVID‐19 treatment, neutralizing antibodies/Nbs are particularly useful for prevention. In this regard, the excellent physicochemical properties of Nb4 may contribute to its strikingly excellent performance in intranasal administration tests. This Nb4 and its multivalent forms can be used as a potent prophylactic and therapeutic agents for all major Omicron variants, including XBB.1.16 and EG.5.

## 4 MATERIALS AND METHODS

### 4.1 Recombinant protein production

#### 4.1.1 Expression and purification of RBD1

The gene of Omicron RBD1 was codon optimized and synthesized by Tsingke Biotechnology Co., Ltd. and subcloned into a modified pFastBac vector, containing the signal peptide of GP64, RBD1 (319‐514) domain, Gly‐Thr linker, Avi‐tag, Gly‐Ser linker, and His_6_ tag. The baculoviruses were prepared as the manufacturer's protocol and infected into the insect cell of *Trichoplusia ni High Five* (*Hi5*) for expression. After 48 h, the supernatant with secreted RBD1 was collected by centrifuging at 1500× *g* for 15 min, and the following reagents were added to a final concentration of 20 mM Tris–HCl pH 8.0, 20 mM imidazole, 1 mM Ni_2_SO_4_, 2 mM CaCl_2_, 150 mM NaCl, and stirred for 30 min at 4°C. The mixture was centrifuged at 15,670× *g* for 30 min at 4°C to collect the supernatant, which was then incubated with the equilibrated Ni‐NTA beads. After batch binding for 2 h, the beads were packed into a gravity column, washed using wash buffer of 30 mM imidazole in buffer A (20 mM Tris–HCl pH 8.0, 150 mM NaCl) for 10 column volume (CV), and eluted of RBD1 using elution buffer of 300 mM imidazole in buffer A. RBD1 was further biotinylated on the Avi tag using MBP‐BirA enzyme, and purified using Superdex 200 Increase 10/300 GL. Purified biotinylated RBD1 was used for phage screening and BLI assay. Aliquots of the purified RBD1 without biotinylation were frozen in liquid nitrogen and stored at −80°C for crystallization with Nbs.

#### 4.1.2 Expression and purification of ACE2

The extracellular ACE2 gene was codon optimized and subcloned into pCAGGS vector, which contained the Kozak sequence, the signal peptide of wild‐type ACE2, ACE2(20‐615), Gly‐Thr linker, Avi tag, Gly‐Ser linker, and His_8_ tag.^6^ The plasmids and PEI 25K (1:3, w/w) were separately diluted using medium, mixed together and transfected into HEK293F for secreting expression at 37°C for 3 days. After that, the collected supernatant plus 20 mM imidazole was added to the equilibrated Ni‐NTA beads (Qiagen), and batch binding was performed for 2 h at 4°C with stirring. The beads were packed into a gravity column and washed using wash buffer of 20 mM imidazole in buffer A, and eluted using elution buffer of 300 mM imidazole in buffer A. The eluted ACE2 was concentrated and further purified into PBS (pH 7.4) with Superdex 200 Increase 10/300 GL. Finally, the protein was frozen in liquid nitrogen and stored at −80°C for further use. Alternatively, some of the ACE2 proteins were biotinylated and utilized for biochemical analysis.

#### 4.1.3 Expression and purification of Nbs

Nbs were subcloned into pSB vector containing a pelB signal peptide, Nbs sequence, myc and His_6_ tag, and transformed into *Escherichia coli* MC1061 strain for expression. The overnight cultured cells were seeded into Terrific Broth medium (1% [w/v] tryptone, 2% [w/v] yeast extract, 0.4% [v/v] glycerol in PBS buffer) at a ratio of 1:100 (v/v) and grown for 2 h to reach an OD_600_ of ∼0.5 in a shaker at 37°C. After the temperature was lowered to 22°C, the cells were cultured for another 1.5 h, and 0.02% (w/v) arabinose was added to induce the expression for 16 h. Cells collected from 100 mL of culture were resuspended using 4 mL of hypertonic buffer (0.2 M Tris–HCl, pH 8.0, 0.5 M sucrose, and 0.5 mM Ethylene Diamine Tetraacetic Acid [EDTA]) and incubated for 0.5 h. Then, 8 mL of cooled MilliQ water was added and mixed immediately to rotate for another 1 h. After centrifuging for 30 min at 20,000× *g*, the supernatant was incubated with 200 μL of Ni‐NTA resin in the presence of 150 mM NaCl, 2 mM MgCl_2_, and 20 mM imidazole for 1.5 h at 4°C. The beads were packed into a gravity column, washed using 30 mM imidazole in buffer A to remove containments, and eluted using 300 mM imidazole in buffer A. Desalting column PD10 (Bio‐Rad) was used to desalt the Nbs into PBS for later usage.

For Nb4‐Fc fusion protein and multivalent Nbs, the expression and purification procedures were similar to those of ACE2 and Nb monomers, respectively. Spike proteins of SARS‐CoV‐2 Omicron were obtained as described in our previous work.^6^

Endotoxin removal from the Nbs used for the mouse assay was carried out following the manufacturer's protocol (Thermo, Pierce High Capacity Endotoxin Removal Spin Column, 0.5 mL, Cat. 88274). Then, aliquots of the resultant Nbs were frozen using nitrogen liquid, and stored at −80°C.

#### 4.1.4 Nb screening by phage panning

Nbs targeting SARS‐CoV‐2 Omicron RBD (RBD1) were selected by phage panning from a synthetic Nbs phage library covering ∼10^9^ members. In brief, 100 μL of SA‐coated magnetic beads (SA beads, Pierce, Cat. 21925) were incubated with phages used for panning to remove the bead‐bounded phages. The unbound phages were incubated with biotinylated RBD1 (∼700 nM) for 1.5 h at room temperature (RT) to form the RBD1–phage complex. The mixture was then incubated with another 100 μL of SA‐coated magnetic beads for 30 min at RT to immobilize the RBD1–phage complex, by which the RBD1‐unbound phages would be more easily removed. After that, the beads were washed using 500 μL of PBS supplemented with 0.1% (v/v) Tween 20 (PBST) buffer while rotating at RT for 10 min. After washing three times, the RBD1‐bound phages were eluted using 0.2 M glycine–HCl (pH 2.2), and the pH of the eluent was adjusted to neutral using 2 M Tris–HCl buffer (pH 8.0). The resultant phages were infected into XL1‐Blue cells and amplified with the helper phage of VCSM13. As for the second‐round panning, the first panning procedures were repeated to consolidate the enriched phages. For the following rounds, the procedures were similar except for the reducing concentration of biotinylated RBD1 (100 nM for rounds 3 and 4 and 10 nM for round 5) and volume of the SA‐beads (from 200 to 100 μL).

#### 4.1.5 Phage enzyme‐linked immunosorbent assay

To evaluate the enrichment of RBD1‐binding phages, a polyclonal phage ELISA was performed. Here, the Nbs selected from each round of the panning were displayed on the surface of the phages. The phages, immobilized on the plates by RBD1, were detected by an antibody against M13 expressed on the tips of the phages. For this, a 96‐well plate was coated with 50 μL of 2 μg/mL RBD1 in PBS for each well and incubated at 4°C overnight. The following day, the plates were washed three times using 100 μL of PBS, blocked with 100 μL of 1% (w/v) milk–PBST (0.05% [v/v] Tween 20) for 1 h at RT, and washed three times using 100 μL of PBST. After that, 10^9^ phages from each panning round were added into each well with three replicates and incubated for 1.5 h at RT. The wells were washed three times using 100 μL of PBST before adding 50 μL of a 1:5000 dilution anti‐M13 bacteriophage antibody conjugated with horseradish peroxidase (HRP) in PBST and incubated at RT for 1 h. The substrate 2,2'‐AZINO‐bis‐(3‐ethylbenziazoline‐6‐sulfonic acid) [ABTS] of 50  μL was added to each well for color development after washing 96‐well plates three times with PBST. The absorbance at 415 nm was recorded after the appropriate incubation time.

To determine whether the selected single colony with unique Nb gene binds to antigen RBD1 or not, the mono‐colony ELISA was carried out. Briefly, a single colony was inoculated into a well with 2YT medium supplemented with appropriate biotics and cultured at 37°C with shaking at 1000 rpm in the 96‐well formats. Once the culture reached an OD_600_ of ∼0.6, helper phage of VCSM13 was added. The culture medium containing secreted phages was collected by centrifuging at 3220× *g* for 20 min at RT, and the supernatants were directly used for ELISA. Except for the unpurified phages used, the remaining procedures were similar to those used for polyclonal ELISA.

#### 4.1.6 Biolayer interferometry assay

BLI experiments were carried out at 25°C using the Octet RED96 machine (FortéBio). All of the sensors were first dipped into equilibration buffer (20 mM HEPES, pH 7.4, 150 mM NaCl, and 10 mg/mL bovine albumin [BSA]) for at least 10 min.

To assess the interaction between purified Nbs and RBD1, streptavidin biosensors were utilized to immobilize biotinylated RBD1 for 60 s. Subsequently, the biosensors were exposed to 500 nM of the Nbs being tested.

For the competitive binding test between Nbs and hACE2 to RBD1, 100 nM biotinylated RBD1 was immobilized onto streptavidin biosensors for 60 s, and then the sensors were saturated with 500 nM tested Nb for 420 s and then dipped into an equal molar concentration of the corresponding Nb with or without 500 nM soluble ACE2 for 300 s.

To determine the binding affinities, biotinylated monovalent, bivalent or trivalent Nb4 was immobilized on streptavidin biosensors, and then the biosensors were dipped into different SARS‐CoV‐2 variant proteins at various concentrations as indicated in the figures.

#### 4.1.7 Neutralization assay using authentic SARS‐CoV‐2 virus

### 4.2 Focus‐forming assay for SARS‐CoV‐2 quantification of virus stock

The SARS‐CoV‐2 wild type, Alpha (B.1.1.7), Beta (B.1.351), Delta (B.1.617.2), and Omicron (BA.1.1, BA.2.3, and BA.5) strains were isolated from COVID‐19 patients, validated by next‐generation sequencing, and restored in Guangzhou Customs District Technology Center BSL‐3 Laboratory. Vero E6 cells pre‐seeded in cell plates were incubated with a serially diluted SARS‐CoV‐2 stock of 50 μL for 1 h at 37°C and changed to 100 μL of minimum essential medium containing 1.2% (v/v) carboxymethylcellulose for further incubation. After 24 h, cells were fixed using 4% (v/v) paraformaldehyde. For permeabilization and staining, cells were sequentially treated with 0.2% (v/v) Triton X‐100, SARS‐CoV/SARS‐CoV‐2 Nucleocapsid Rabbit mAb (Sino Biological, 40143‐T62) and Peroxidase AffiniPure Goat Anti‐Rabbit IgG (H+L) (Jackson, 111‐035‐144). After that, virus foci were visualized using KPL TrueBlue Peroxidase substrate (Seracare Life Science, 5510‐0030), counted and analyzed using a CTL ImmunoSpot S6 Ultra analyzer (Cellular Technology Limited). Virus titers were determined according to the number of virus foci and their dilution.

### 4.3 Focus reduction neutralization test

For neutralization capacity determination, an equal volume of SARS‐CoV‐2 (200 FFU) and 50 μL of serially diluted Nbs were co‐incubated for 1 h at 37°C. Then, the mixtures of 50 μL were distributed to the cell plates pre‐seeded with Vero E6 cells. Following this, the procedures of the FFA method were performed. IC_50_ values corresponding to 50% of the focus reduction neutralization test titer were determined and calculated by GraphPad Prism.

### 4.4 Crystallization and structure determination of RBD1‐Nb4 complex

The RBD1 and Nb4 proteins were mixed at a molar ratio of 1:2 and further purified by Superdex 200 Increase 10/300 GL column after incubation on ice for 2 h. Crystal screening of the RBD1‐Nb4 complex at 5 mg/mL was performed by vapor‐diffusion hanging‐drop method at RT. Diffracting crystals were obtained in the condition of 0.2 M potassium thiocyanate and 20% (w/v) polyethylene glycol 3350 (PEG 3350).

A diffraction data set with 2.43 Å was collected at BL19U1 beamline in the National Center for Protein Science Shanghai (NCPSS) and processed with HKL3000 software.^41^ The RBD1‐Nb4 complex structure was determined by the molecular replacement method using Phenix^42^ with previously reported SARS‐CoV‐2 RBD structure (PDB: 6M0J) and Nb structure (PDB: 7TE8), and the atomic models were further refined and completed using Phenix^42^ and Coot,^43^ respectively. Data collection, processing, and refinement statistics are summarized in Table S2. PyMOL (http://www.pymol.org) was used to generate the structural model figures.

### 4.5 Cryo‐EM sample preparation and data processing

Mix 1.2 mg/mL of purified SARS‐CoV‐2 Omicron BA.1 and BA.5 spike proteins with Nb4‐30t or Nb4‐16t Nbs at a 1:3 molar ratio in PBS (pH 7.4) buffer. Three microliters of protein sample was applied onto the copper grid (300‐mesh Quantifoil R1.2/1.3), which was glow‐discharged using H_2_/O_2_ method (Quantifoil, Micro Tools GmbH). The grid was blotted with a blot force of 0 for 3 s at 8°C and 100% humidity and then plunge‐frozen into liquid ethane using a Vitrobot (Thermo Fisher Scientific).

A 300 kV Titan Krios microscope (Thermo Fisher Scientific) equipped with a K3 detector (Gatan) was used for Cryo‐EM dataset collection. The exposure time was set to 2 s, and the total accumulated dose was 60 electrons/Å^2^, which yields a final pixel size of 0.82 Å. A total of 3988 micrographs were collected in a single session using SerialEM for the complex of Omicron BA.1 Spike and Nb4‐30t with a defocus range comprised between 1.2 and 2.2 μm, while 4509 micrographs were collected in a single session for the complex of Omicron BA.5 Spike and Nb4‐16t. The statistics of Cryo‐EM data collection can be found in Table S3.

Motion correction and dose weighting for all dose‐fractioned images were performed by MotionCorr2 software,^44^ and the contrast transfer functions were estimated by cryoSPARC patch CTF estimation.^45^

For the dataset of Omicron BA.1 Spike and Nb4‐30t complex (Figure S6D), a total of 1,898,557 particles were auto‐picked using the template picker job and 971,923 raw particles were extracted with a box size of 512 pixels. A total of 208,758 particles were left after 2D classification and used to perform ab initio reconstruction in six classes. Then, these classes were used as 3D volume templates for heterogeneous refinement with all selected particles, with 118,567 particles converging into a “2‐up” conformation bound to two Nb4 on an “up” RBD and a “down” RBD. Next, the particle set was used to perform non‐uniform refinement, yielding a resolution of 3.6 Å. The above 2D and 3D classifications and refinements were all performed using cryoSPARC.

For the dataset of Omicron BA.5 Spike and Nb4‐16t complex, a similar process was performed as above (detailed in Figure S6A). Finally, 60,877 particles converged into mono‐bound Omicron Spike–Nb4 “2‐up/1‐down” conformation, and 40,530 particles converged into triple‐bound Omicron Spike–Nb4 “2‐up/1‐down” conformation. The two particle sets were applied to perform nonuniform refinement, yielding a 3.3 Å resolution for mono‐bound Omicron Spike–Nb4 complexes and a 3.5 Å for triple‐bound Omicron Spike–Nb4 complexes.

### 4.6 Cryo‐EM model building and refinement

To build the structures of the SARS‐CoV‐2 Omicron BA.5 Spike–Nb4 complex, we used the recently reported SARS‐CoV‐2 Omicron BA.5 variant spike Cryo‐EM structure^46^ (PDB: 7XNQ) as an initial model with additional parts of the Nb4 and RBD1 model taken from previously described Swiss structure. Then manual docking of the models was performed in ChimeraX 1.4^47^ with the guidance of the Cryo‐EM electron density maps. Phenix 1.20^48^ and Coot 0.9^43^ were used to perform overall real‐space refinements. The data validation statistics are shown in Figure S6B,C and Table S3.

To build the structures of the SARS‐CoV‐2 Omicron BA.1 Spike–Nb4 complex, a similar workflow was carried out as described above except that the initial model was replaced with SARS‐CoV‐2 Omicron variant Spike‐510A5 Fab Cryo‐EM structure^6^ (PDB: 7WS4) and our crystal structure (PDB: 8K3K). The data validation statistics are shown in Figure S6E and Table S3.

### 4.7 Sample preparation and lyophilization test

One hundred microliters of 1 mg/mL Nb4 in 1× PBS (pH 7.4) was transferred into a 1.5 mL EP tube and lyophilized using the freeze‐drying cycle method in a VirTis AdVantage machine. After that, the powder was stored at −80°C. To measure its biochemical characteristics, the powder was re‐dissolved in 1× PBS (pH 7.4).

### 4.8 Nanoscale differential scanning fluorometry assay

The thermal stability of fresh Nb4 and re‐dissolved lyophilized Nb4 was determined by nanoscale differential scanning fluorometry (Prometheus NT.48). Ten microliters of 0.01 mg/mL Nb4 in 1× PBS (pH 7.4) was filled into the capillaries and placed into the sample holder for monitoring its thermal unfolding with 1°C/min thermal ramp from 20°C to 95°C.

### 4.9 SARS‐CoV‐2 challenge experiment

Specific pathogen‐free 5–6‐week‐old female BALB/c mice were purchased from Guangdong GemPharmatech and maintained in the Animal Care Facilities at the Guangzhou Medical University. Experiments were performed following the protocols approved by the Institutional Animal Care and Use Committees of the Guangzhou Medical University. The mouse challenge experiment was carried out in Guangzhou Customs District Technology Center BSL‐3 Laboratory. Mice in the prophylactic group were pre‐administrated with 200 μg (10 mg/kg, i.p.) or 75 μg (3.75 mg/kg, i.n.) of Nbs 3 h before challenge with 1 × 10^5^ FFU virus (BA.1.1 or BA.2.3), while mice in the therapeutic group were administrated with 200 μg (10 mg/kg, i.p.) 2 h after virus challenge. Lung tissue was harvested for virus titering on day 1 post‐infection and harvested for hematoxylin–eosin staining (*n* = 3 per group) on day 3 post‐infection. The virus titer was determined by a FFA. The experimental design model in Figure 3 was created with Figdraw (ID: ARUAYefafa). GraphPad Prism was used for data analysis and plotting.

## AUTHOR CONTRIBUTIONS

H.Y. designed and performed the phage display screening assay, purified selected Nbs, and characterized them using BLI assay with assistance from Zhiying Z. and Y.Z. H.Y. generated all plasmids, purified Nbs, and some RBDs for all follow‐up mechanistic, crystallization, Cryo‐EM, and functional experiments. The crystal structure was also analyzed by H.Y. H.W. purified ACE2 and Nb4‐Fc proteins, performed binding kinetics and competition assays, crystallized and determined crystal structure of the RBD1–Nb4 complex, and tested Nb stabilities. Zhaoyong Z. and X.X. evaluated. J.Z. and Y.W. conceived and designed the protection capacity of Nbs in vitro and in vivo. Y.L. under supervision of Haitao Y. solved Cryo‐EM structures of BA.1/Nb4 and BA.5/Nb4 complexes. H.Y., Z.W., and W.X. drafted the manuscript with inputs and revisions from all co‐authors. All authors have read and approved the final manuscript.

## CONFLICT OF INTEREST STATEMENT

H.Y., W.X., H.W., Zhiying Z., Y.Z., Z.W., Y.L., and Haitao Y. filed patents on Nb4 nanobody and the application for COVID‐19 treatment and prevention and the PCT number is PCT/CN2022/084209. Zhaoyong Z., X.X., Y.W., and J.Z. declare that they have no conflicts of interest.

## ETHICS STATEMENT

Animals experiments were accorded with the protocols approved by the Institutional Animal Care and Use Committees of Affiliated First Hospital of Guangzhou Medical University (approval number: 20230325).

## Supporting information

Supporting information

## Data Availability

The atomic coordinates and crystallographic structure factors of RBD1–Nb4 complex generated in this study have been deposited in the PDB under the accession code 8K3K. Cryo‐EM reconstructions and atomic models generated in this study are available under the accession codes PDB (EMDB) IDs 8K47 (EMD‐36879), 8K46 (EMD‐36878), and 8K45 (EMD‐36877). The corresponding author will provide the relevant data upon request.
