# Supplementary material for: A potent and broad‐spectrum neutralizing nanobody for SARS‐CoV‐2 viruses, including all major Omicron strains
Source: MedComm (2020). 2023 Oct 26;4(6):e397. doi: 10.1002/mco2.397 (PMC10600506; doi:10.1002/mco2.397)

**A potent and broad-spectrum neutralizing nanobody for SARS-CoV-2 viruses including all major Omicron strains**

Running title: A nanobody neutralize all major Omicron variants

Hebang Yao^1,#^, Hongyang Wang^1,#^, Zhaoyong Zhang^2,#^, Yuchi Lu^1,3,#^, Zhiying Zhang^1,^, Yu Zhang^1,^, Xinyi Xiong^1,^, Yanqun Wang^2,4,^, Zhizhi Wang^1,^, Haitao Yang^1,3,^*, Jincun Zhao^2,3,5,6,7,^*, Wenqing Xu^1,^*

^1^ School of Life Science and Technology, ShanghaiTech University, Shanghai, China

^2^ State Key Laboratory of Respiratory Disease, National Clinical Research Center for Respiratory Disease, Guangzhou Institute of Respiratory Health, the First Affiliated Hospital of Guangzhou Medical University, Guangzhou, Guangdong 510182, China

^3^ Shanghai Institute for Advanced Immunochemical Studies, ShanghaiTech University, Shanghai, China

^4^ GMU-GIBH Joint School of Life Sciences, Guangzhou Medical University, Guangzhou, Guangdong, China.

^5^ Guangzhou Laboratory, Bio-Island, Guangzhou, Guangdong, China.

^6^ Institute of Infectious disease, Guangzhou Eighth People's Hospital of Guangzhou Medical University, Guangzhou, Guangdong, China.

^7^ Institute for Hepatology, National Clinical Research Center for Infectious Disease, Shenzhen Third People's Hospita, Shenzhen, Guangdong, China.

^#^ Contributed equally to this work.

* Corresponding authors: yanght@shanghaitech.edu.cn (Haitao Y.), zhaojincun@gird.cn (J.Z.), [xuwq2@shanghaitech.edu.cn](mailto:xuwq2@shanghaitech.edu.cn) (W.X.)

## Supplementary information

**Supplementary information, Figure S1 The binding affinities of Nbs selected from mono-ELISA assay with RBD1.** Interactions between purified Nbs and RBD1 were confirmed using BLI assay with 500 nM Nbs as the analyte. The biotinylated RBD1 of 5 μg/mL was immobilized on the SA sensor.


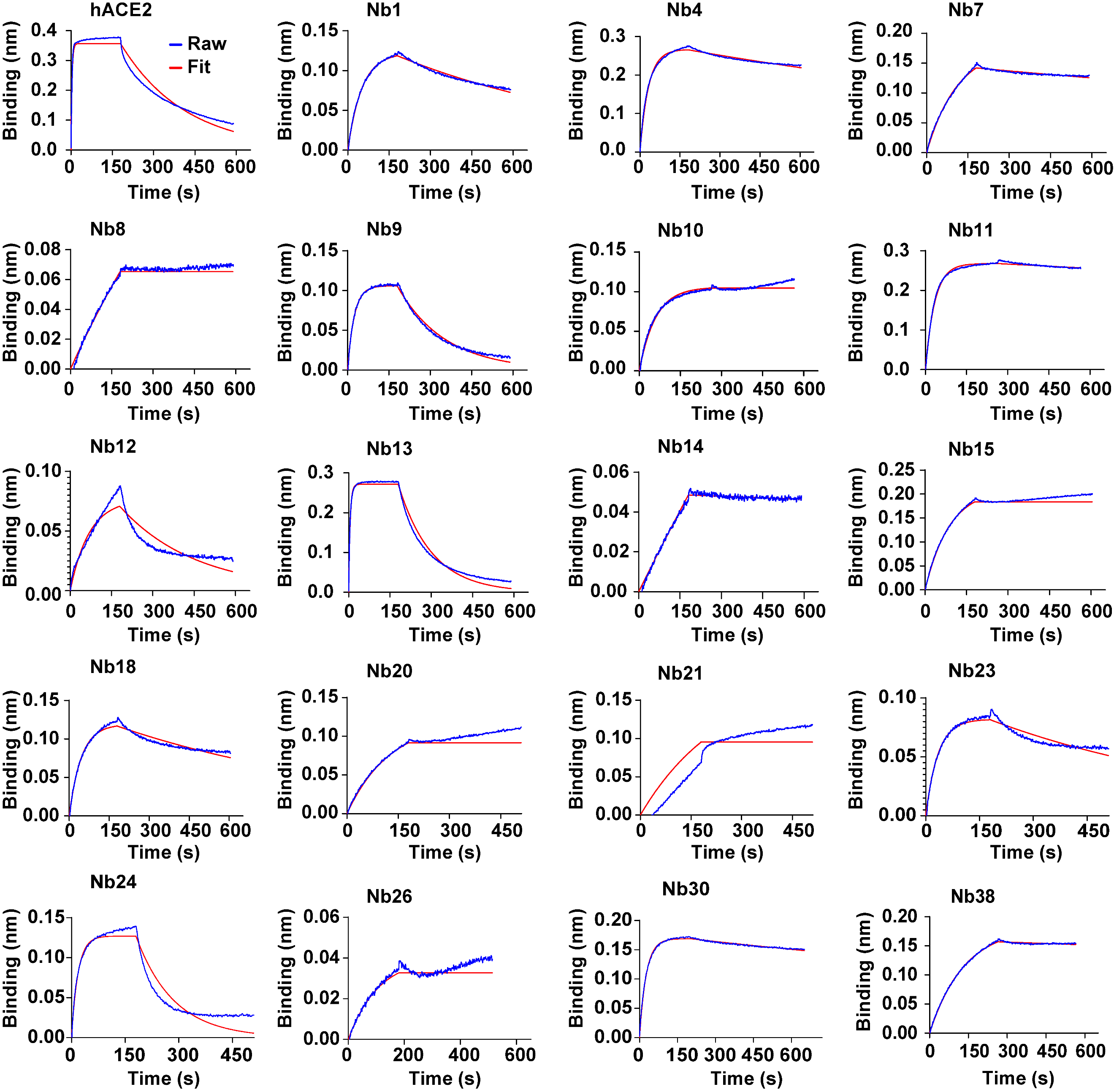


**Table S1 Summary of the kinetics parameters in the Figure S1.**

| Nbs | *K*_D_ (nM) | *k*_on_ (1/Ms) | *k*_off_ (1/s) | X^2^ | R^2^ |
| --- | --- | --- | --- | --- | --- |
| Nb1 | 32 | 3.7*10^4^ | 1.2*10^-3^ | 0.0026 | 0.99 |
| Nb4 | 7.4 | 6.2*10^4^ | 4.6*10^-4^ | 0.0093 | 0.99 |
| Nb7 | 17 | 1.7*10^4^ | 3.0*10^-4^ | 0.0026 | 0.99 |
| Nb8 | N.R. | 2.9*10^3^ | < 1.0*10^-7^ | 0.0037 | 0.98 |
| Nb9 | 80 | 7.2*10^4^ | 5.8*10^-3^ | 0.0053 | 0.99 |
| Nb10 | < 1 pM | 3.4*10^4^ | < 1.0*10^-7^ | 0.0079 | 0.97 |
| Nb11 | 2.3 | 5.9*10^4^ | 1.4*10^-4^ | 0.0066 | 0.99 |
| Nb12 | 166 | 2.2*10^4^ | 3.6*10^-3^ | 0.0287 | 0.85 |
| Nb13 | 24 | 3.5*10^5^ | 8.2*10^-3^ | 0.074 | 0.99 |
| Nb14 | 200 | 5.3*10^2^ | 1.1*10^-4^ | 0.001 | 0.99 |
| Nb15 | < 1 pM | 2.3*10^4^ | < 1.0*10^-7^ | 0.0294 | 0.97 |
| Nb18 | 24 | 4.2*10^4^ | 1.0*10^-3^ | 0.0092 | 0.96 |
| Nb20 | < 1 pM | 1.6*10^4^ | < 1.0*10^-7^ | 0.0367 | 0.89 |
| Nb21 | < 1 pM | 8.3*10^3^ | < 1.0*10^-7^ | 0.2012 | 0.77 |
| Nb23 | 25 | 5.7*10^4^ | 1.4*10^-3^ | 0.006 | 0.94 |
| Nb24 | 100 | 9.1*10^4^ | 9.5*10^-3^ | 0.0733 | 0.93 |
| Nb26 | N.R. | 1.9*10^4^ | < 1.0*10^-7^ | 0.0053 | 0.88 |
| Nb30 | 3.8 | 7.6*10^4^ | 2.9*10^-4^ | 0.0017 | 0.99 |
| Nb38 | 6.8 | 1.5*10^4^ | 1.0*10^-4^ | 0.0011 | 0.99 |

**Supplementary information, Figure S2 SDS-PAGE analysis of the samples for neutralization and mice challenge assay.** Molecular weight marker (kDa) was labeled on the left.


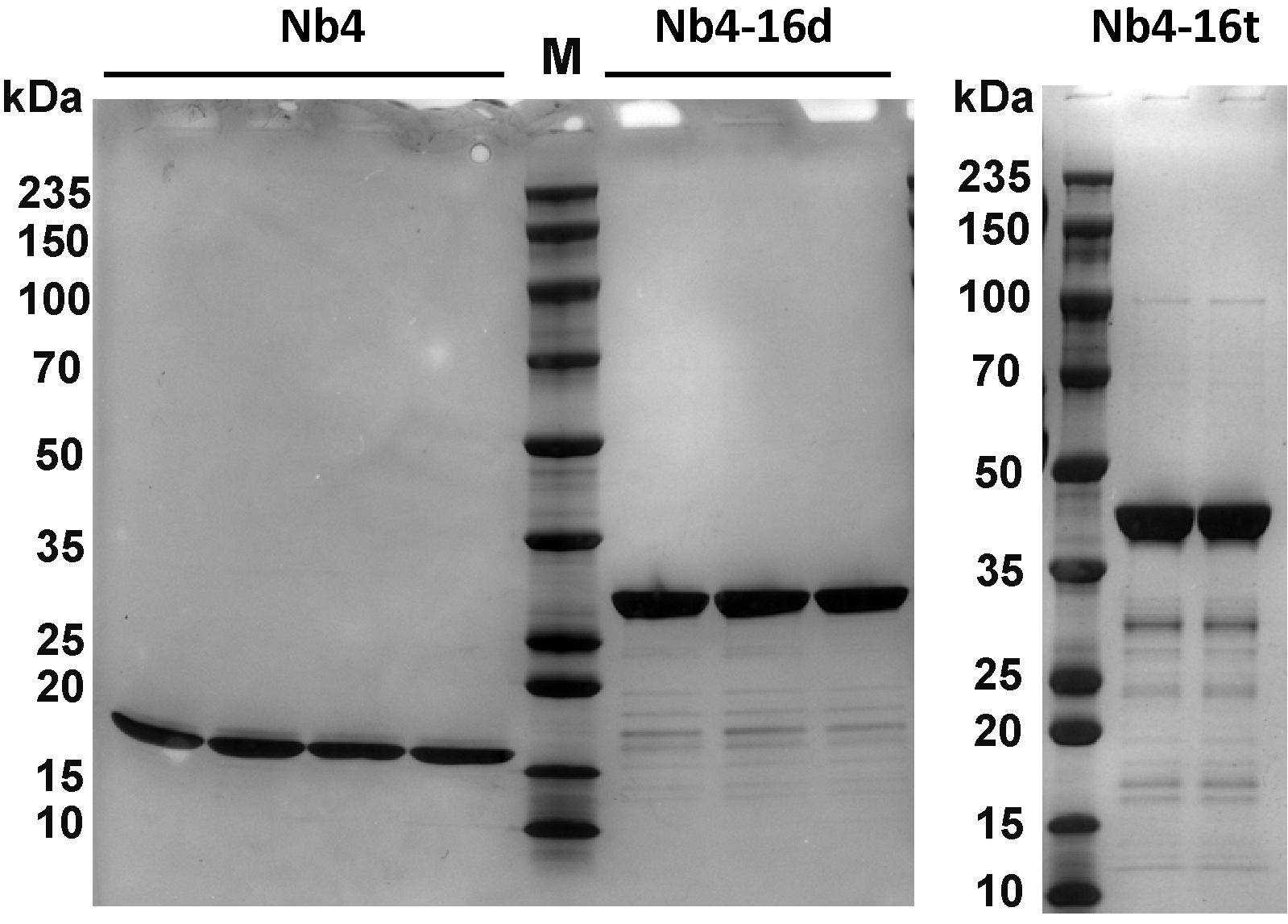


**Supplementary information, Figure S3 Binding characteristic between multivalent Nb4 and RBD1 or BA.2 spike ECD protein (Spike 2). (A, B)** Binding kinetics determination of RBD1 with ACE2 or multivalent Nb4 (A), Spike 2 with Nb4 or multivalent Nb4 (B). The concentration (nM) of analyte and kinetics parameters were shown as indicated.


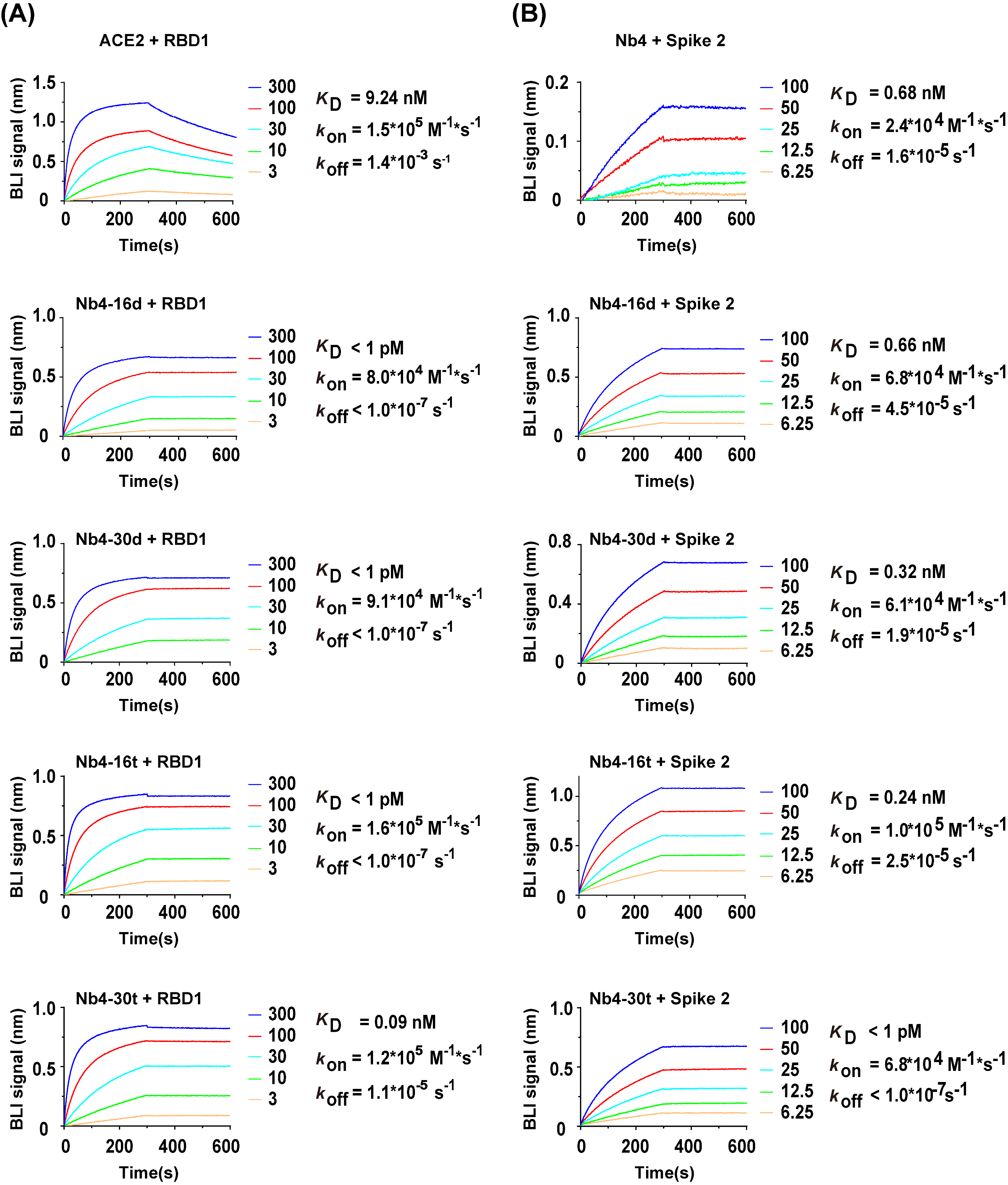


**Supplementary information, Figure S4 Neutralization assay of Nb4-30d to Delta strains. (A, B)** BLI binding assay of Nb4-16t with WT spike (A) or Delta RBD (B). **(C)** Neutralization ability determination of Nb4-30d and Nb4-16t to SARS-CoV-2 delta strain. **(D)** RBD sequence alignment between SARS-CoV-2 WT and Delta strain.


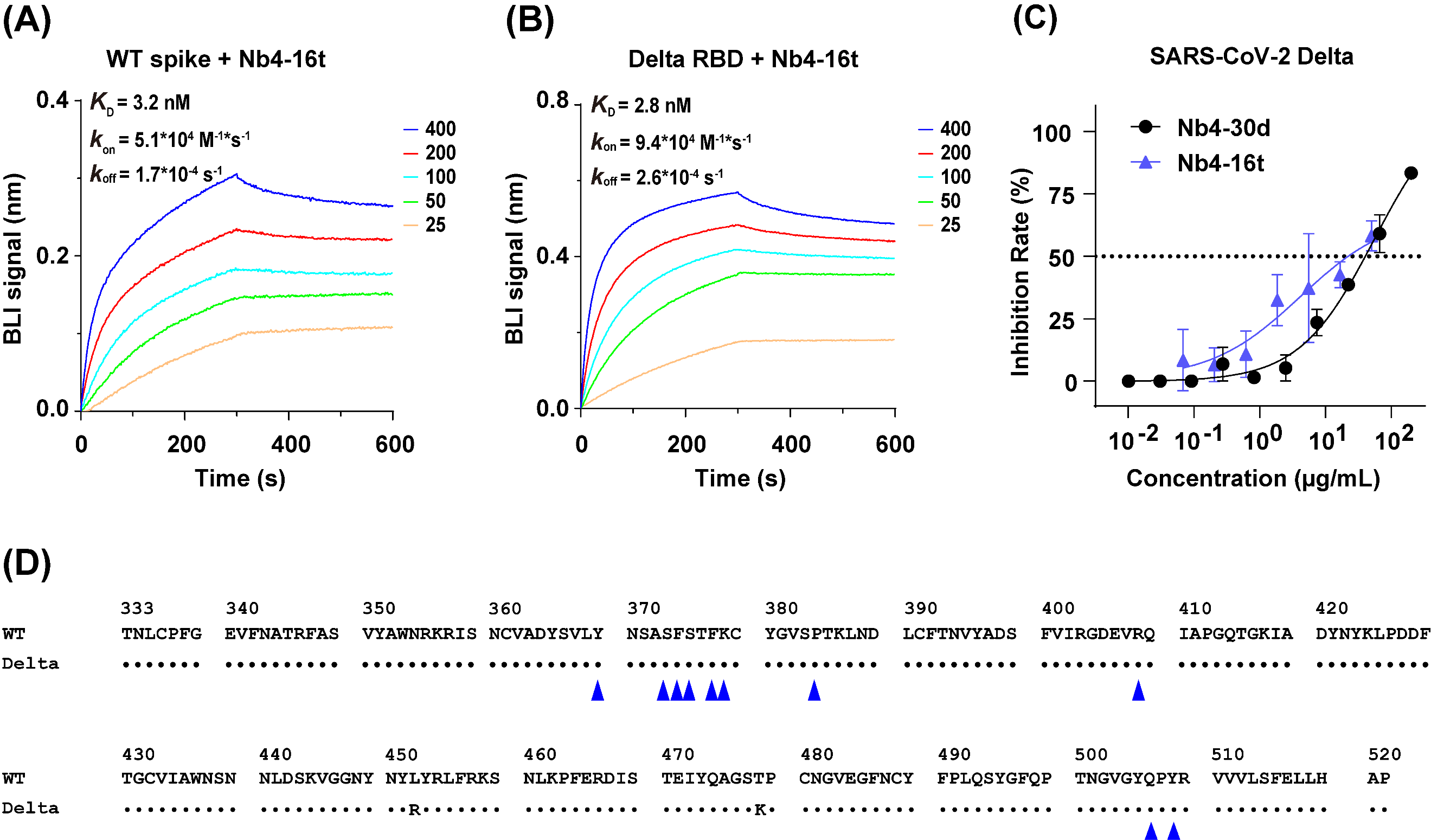


**Table S2 X-ray Data collection and refinement statistics**

|  | RBD1 + Nb4 (PDB code: 8K3K) |
| --- | --- |
| **Data collection** |  |
| Space group | *P*6_1_22 |
| Cell dimensions |  |
| *a, b, c* (Å) | 76.11, 76.11, 251.45 |
| *α, β, γ* (°) | 90, 90, 120 |
| Wavelength (Å) | 0.9792 |
| Resolution (Å) | 35.37-2.43 (2.49-2.43) |
| R_pim_ (%) | 3.2 (48.0) |
| *I* / σ*I* | 14.8 (1.7) |
| CC1/2 | 0.997 (0.706) |
| Completeness (%) | 100.0 (100.0) |
| Redundancy | 36.8 (37.3) |
| **Refinement** |  |
| Resolution (Å) | 35.37-2.43 |
| No. of reflections | 30440 |
| No. of water | 19 |
| R_work_/R_free_^a,b^ (%) | 22.40/26.92 |
| No. atoms | 2677 |
| Average B factor (Å^2^) |  |
| Protein | 81.86 |
| H_2_O | 63.43 |
| R.m.s deviations |  |
| Bond lengths (Å) | 0.003 |
| Bond angles (°) | 0.633 |
| Ramachandran plot (%) |  |
| Most Favorable | 93.39 |
| allowed | 6.61 |
| Outliers | 0 |

Values in parentheses are for highest-resolution shell.

^a^ *R*_work_ = Σ_hkl_ |Fo(hkl) − Fc(hkl)|/Σ_hkl_ Fo(hkl).

^b^ *R*_free_ was calculated for a test set of reflections (5%) omitted from the refinement.

**Table S3 Cryo-EM data collection, models refinement and validation statistics of the Omicron Spike-Nb4 datasets.**

|  | BA.5 + Nb4-16t  mono-bound  “2-up” conformation  PDB, 8K47 | BA.5 + Nb4-16t  triple-bound  “2-up” conformation  PBD, 8K46 | BA.1 + Nb4-30t  “2-up” conformation  PDB, 8K45 |
| --- | --- | --- | --- |
| **Data collection and processing** |  |  |  |
| Voltage (kV) | 300 | 300 | 300 |
| Detector | K3 | K3 | K3 |
| Pixel size (Å) | 0.816 | 0.816 | 0.82 |
| Electron dose (e^-^/ Å^2^) | 60 | 60 | 60 |
| Defocus range | -1.2 to -2.2 | -1.2 to -2.2 | -1.2 to -2.2 |
| Final particles | 40,530 | 60,877 | 118,567 |
| Final resolution (Å) | 3.3 | 3.5 | 3.6 |
| **Model refinement** |  |  |  |
| Map-model CC (mask) | 0.81 | 0.85 | 0.84 |
| Initial model used | 7XNQ | 7XNQ | 7WS4 |
| RMSD |  |  |  |
| Bond lengths (Å) | 0.003 | 0.003 | 0.002 |
| Bond angles (°) | 0.577 | 0.603 | 0.537 |
| Molprobity score | 2.09 | 2.04 | 1.80 |
| Clash score | 12.6 | 11.92 | 9.29 |
| Rotamer outliers (%) | 0.27 | 0.48 | 0.48 |
| Cβ outliers (%) | 0.00 | 0.03 | 0.00 |
| CaBLAM outliers (%) | 5.52 | 4.90 | 3.13 |
| **Ramachandran statistics** |  |  |  |
| Favored (%) | 92.17 | 92.92 | 95.58 |
| Allowed (%) | 7.62 | 6.92 | 4.36 |
| Outliers (%) | 0.21 | 0.17 | 0.06 |

**Supplementary information, Figure S5 Sequences alignment of the different SARS-CoV-2 strains.** Blue triangles show the residues involved in the RBD1 and Nb4 interface.


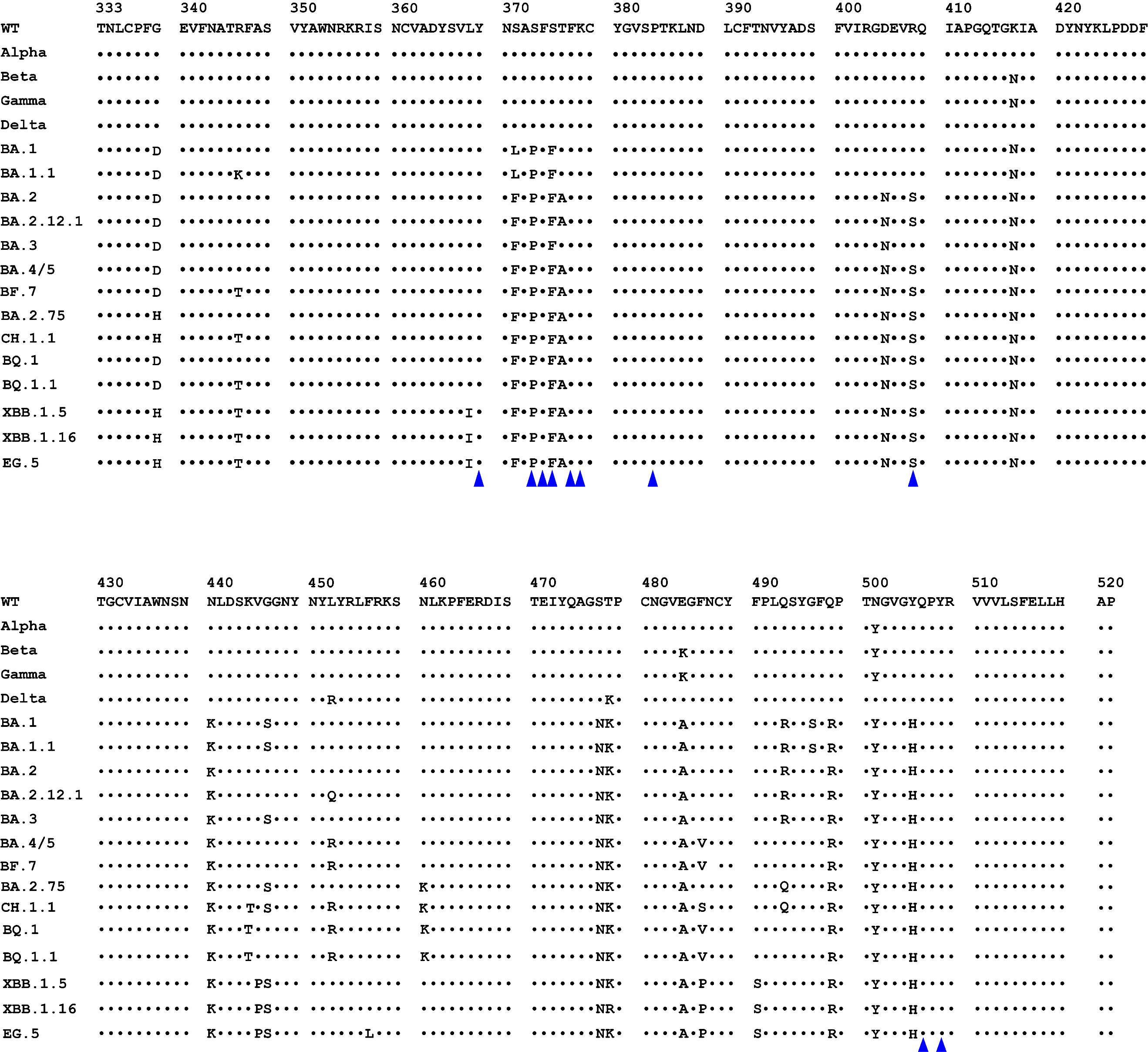


**Figure S6 Cryo-EM data processing for the Omicron Spike protein and nanobody Nb4 complex.**

**(A)** 3D reconstruction workflow of the Omicron BA.5 Spike/Nb4-16t complex. **(B, C)** The viewing direction distribution plot, gold-standard FSC curves with the 0.143 cutoff indicated by a horizontal blue line, Global FSC and Histogram, and Cryo-EM maps colored by local resolution for the a “2-up/1-down” conformation bind with one nanobody (B) or three nanobodies (C), respectively. **(D)** 3D reconstruction workflow of the Omicron BA.1 Spike/Nb4-30t complex. **(E)** The viewing direction distribution plot, gold-standard FSC curves with the 0.143 cutoff indicated by a horizontal blue line, Global FSC and Histogram, and cryo-EM maps colored by local resolution for the a “3-up” conformation bind with two nanobodies.


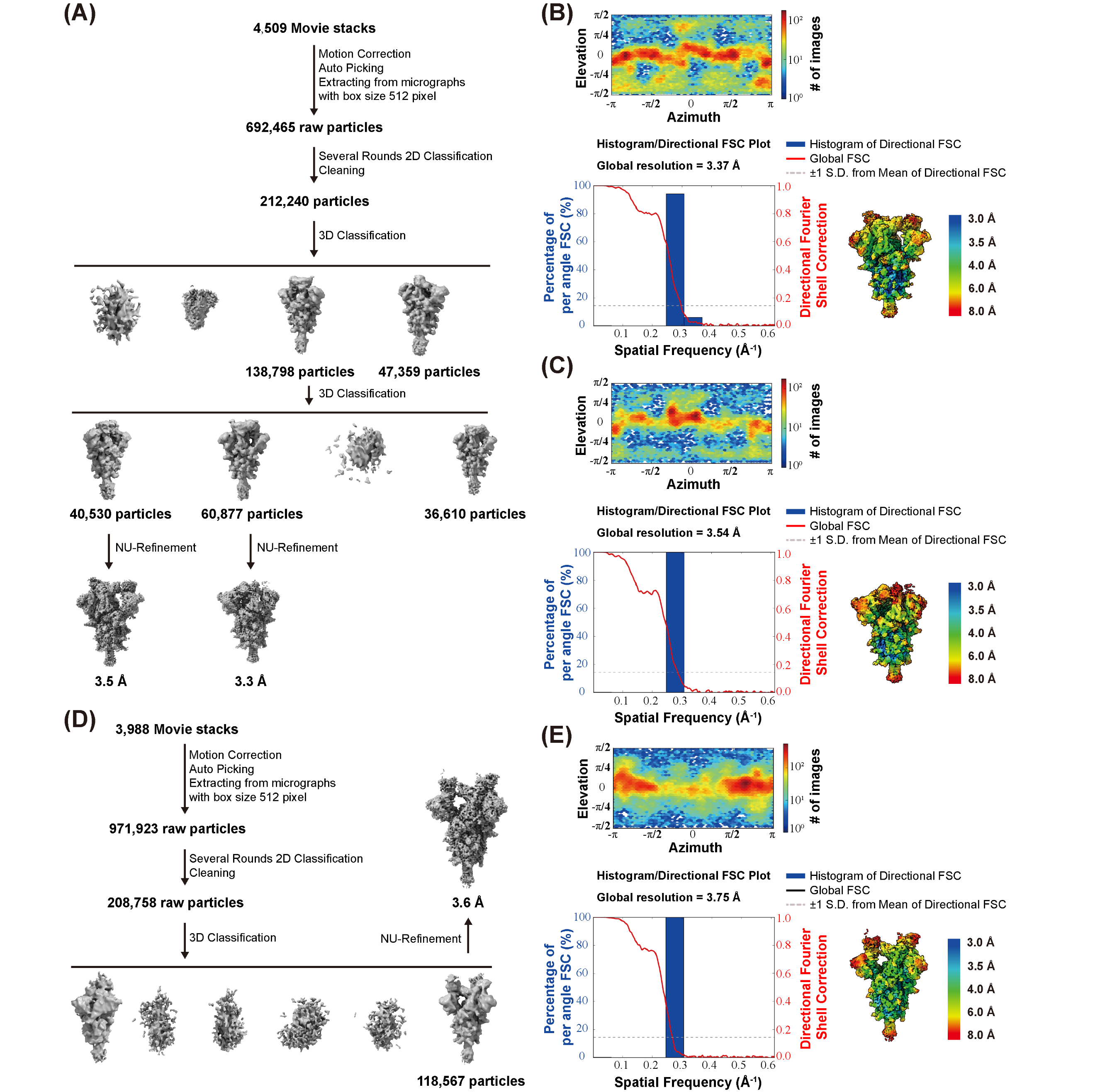

Supplement: Supplementary file 1 — Supporting information [file MCO2-4-e397-s001.docx]
